# Prodrug-based nanomedicines for rheumatoid arthritis

**DOI:** 10.1186/s11671-023-03950-1

**Published:** 2024-01-05

**Authors:** Pei Li, Cong Wang, Hongjie Huo, Chunyun Xu, Huijun Sun, Xinyu Wang, Li Wang, Lei Li

**Affiliations:** College of Pharmacy, Dalian Medical University, Dalian, China

**Keywords:** Rheumatoid arthritis, Prodrug-based nanoparticulate, Drug delivery systems, Targeted therapy

## Abstract

Most antirheumatic drugs with high toxicity exhibit a narrow therapeutic window due to their nonspecific distribution in the body, leading to undesirable side effects and reduced patient compliance. To in response to these challenges, prodrug-based nanoparticulate drug delivery systems (PNDDS), which combines prodrug strategy and nanotechnology into a single system, resulting their many advantages, including stability for prodrug structure, the higher drug loading capacity of the system, improving the target activity and bioavailability, and reducing their untoward effects. PNDDS have gained attention as a method for relieving arthralgia syndrome of rheumatoid arthritis in recent years. This article systematically reviews prodrug-based nanocarriers for rheumatism treatment, including Nano systems based on prodrug-encapsulated nanomedicines and conjugate-based nanomedicines. It provides a new direction for the clinical treatment of rheumatoid arthritis.

## Introduction

Rheumatoid arthritis (RA) is a chronic systemic autoimmune disease which is characterized by clinical symptoms of chronic, symmetric, multiple arthritis and extra-articular lesions [1, 2]. RA affects over 20 million people worldwide and has a high incidence rate of 0.5–1% in the general population [3, 4]. Among patients with RA, the disability related to disease usually appears early in RA patients [5]. RA is a disease in which the immune system incorrectly assaults its own tissues, releasing inflammatory substances that attack the synovial membrane, which is the inner layer of the joint capsule and produces fluid to maintain joints smooth movement [1]. Swollen synovium dilates, causing pain and tenderness in the joint area, as well as redness and difficulty moving the joint. It is relevant to serious bone and cartilage destruction. The clinical manifestations of symmetrical joint involvement are joint pain, swelling, redness, and even limited range of motion [6]. In addition to the joint, many advanced patients also develop systemic and extra-joint diseases, such as coronary heart disease, atherosclerosis, pleurisy, diffuse interstitial pneumonia, etc., significantly reduces life expectancy.

**Fig. 1:**
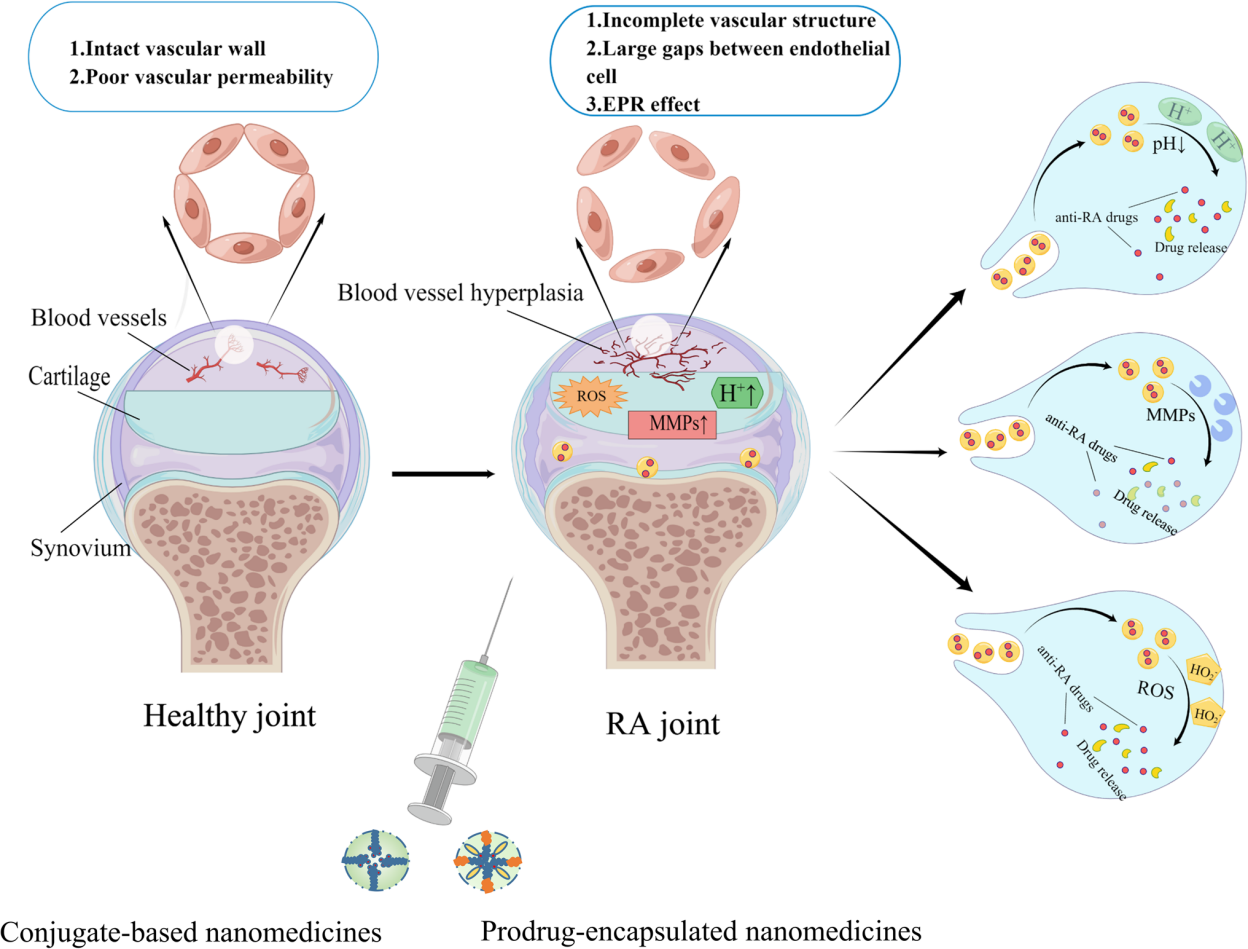
The distinction of microenvironment between healthy joint and RA joint, and the principle of endogenous stimuli-responsive materials releasing drugs

The RA microenvironment (RAM) exerts a profound impact on the pathologic change of rheumatoid arthritis, including synovial hyperplasia, neovascularization, and cartilage and bone wreck [7, 8] (Fig. 1). The present studies reveal that the inflamed synovium in RA would similar properties of “enhanced permeability and retention” (EPR) effect as in solid tumors-like. In addition, RAM contains particular cell types, as well as extracellular matrix molecules which are vitally distinct from those found in healthy sites, including inflammatory factors, reactive oxygen and nitrogen species (RONS), immunogenic proteins, and oxygen [8, 9]. The RAM consists of two distinct components: the extracellular matrix and stromal cells, which exhibit marked differences from those found in healthy tissues [7, 10]. Compared to normal joints, the extracellular matrix of RAM of RA boasts a higher-content of a series of inflammatory factors, matrix metalloproteinases (MMP), many kinds of RONS, H^+^, while simultaneously exhibiting a lower concentration of oxygen [7]. The cfDNA and the immune complex, containing citrullinated protein, incite immune cells secretion of superabundant inflammatory factors [11, 12]. Inflammation engulfs a substantial amount of oxygen, leading to hypoxia and acidic conditions in the 1 system [13], which subsequently triggers the generation of reactive oxygen species (ROS), angiogenesis, and infiltration by inflammatory cells. Simultaneously, inflammatory factors can cause an upsurge in reactive oxygen and RONS concentration within the reticuloendothelial system, and vice versa [14]. A unique vicious cycle is formed by a variety of inflammatory factors and reactive oxygen and RONS, which plays a pivotal role in the progression of RA [15]. These diverse factors of RAM interact mutually, resulting in the relentless progression of RA [7, 16]. In particular, RONS and inflammation mutually influence to give rise to a vicious cycle that drives synovial proliferation, angiogenesis, articular cartilage degradation and bone wreck in RA [17, 18].

There is currently no treatment for rheumatoid arthritis. The treatment goal of RA is to control inflammation and pain, providing patients with symptom relief, and avoiding or minimizing disability by preventing from destructive processes [19]. The therapeutics of RA is dominated by medicines [20], containing non-steroidal anti-inflammatory drugs (NSAIDs), glucocorticoids (GC), disease-modifying antirheumatic drugs (DMARDs) and biological therapeutic agents [21–23] (Table 1). NSAIDs are supposed to extremely alleviate redness, swelling, heat and pain by restraining cyclooxygenase activity. While oral NSAIDs have a short half-life, frequent and high dose dosing is required, which often results in fluid retention, allergies, and renal dysfunction. GCs, such as dexamethasone (Dex) can effectively inhibit inflammatory response and affect the behavior of immune cells. Thus, only high-dose or long-term GCs could manage effective RA treatment. This is worrisome because GCs has a variety of different dose-dependent adverse effects. Methotrexate (MTX), as a prototypical DMARD currently, was initially developed for anti-tumor therapy but later demonstrated remarkable of RA. Nonetheless, in the RA progression stage, DMARDs are often used for the treatment of RA in conjunction with NS efficacy and was deemed a first-line medicines for RA in the 1980s [24]. Compared with else treatments, they can promote the clinical course of RA and alleviate the clinical symptoms of redness, swelling, heat and pain, as well as restrict the progression AIDs or GCs, but have many setbacks, such as oral mucosal inflammation, gingivitis, glossitis, loss of appetite, nausea, vomiting and diarrhea [21–23, 25]. Biotherapeutic agents are used for specific immunotherapy of important cytokines involved in the pathogenesis of RA. The efficacy of biotherapeutic agents is higher than that of conventional methods, but there are still some side effects, such as instability, low bioavailability, high rate of infection, in particular lymphomas [26].

**Table 1:** Current treatment strategies for RA

Classifiction	Drugs	Mechanism of action	Adverse effects
DMARDs	MTX, HCQ, SSZ, LEF	Suppress the immune system	Myelosuppresion, gastrointestinal response
NSAIDs	ASP, CAL, IPF, IDT	Inhibit the synthesis of cyclooxygenase	Gastrointestinal side effects, liver and kidney damage and cardiovascular side effects
GCs	Dex, PN, BUD	Affect the level of inflammatory cytokines regulate immunity	Cause and exacerbate infection, hypertension, and iatrogenic hyperadrenocortical dysfunction
Biological agents	TCZ	Antagonize the IL-6 receptor	Infection, gastrointestinal perforation
	ABA	Downregulate the T cell activation	Infection, hypertension
	AKR	Antagonize the IL-1 receptor	Infection

Most antirheumatic drugs exhibit a narrow therapeutic window, leading to side effects due to their specific distribution in the body. To cope with these challenges, multitude strategies have been conducted, involving prodrug and nanoparticulate drug delivery approaches. Prodrugs, which are inactive compounds that release parent components after metabolism in vivo, have been extensively employed to enhance the effectiveness of existing antirheumatic drugs [27, 28]. A well-executed prodrug strategy can effectively tackle various challenges in drug delivery, such as low solubility, instability, adverse effects, lack of specificity and poor cell uptake. Despite the progress made in conventional prodrug strategies, their clinical application has been greatly restricted by certain flaws. Small molecule prodrugs may be susceptible to quick removal and untimely degradation [29].

**Fig. 2:**
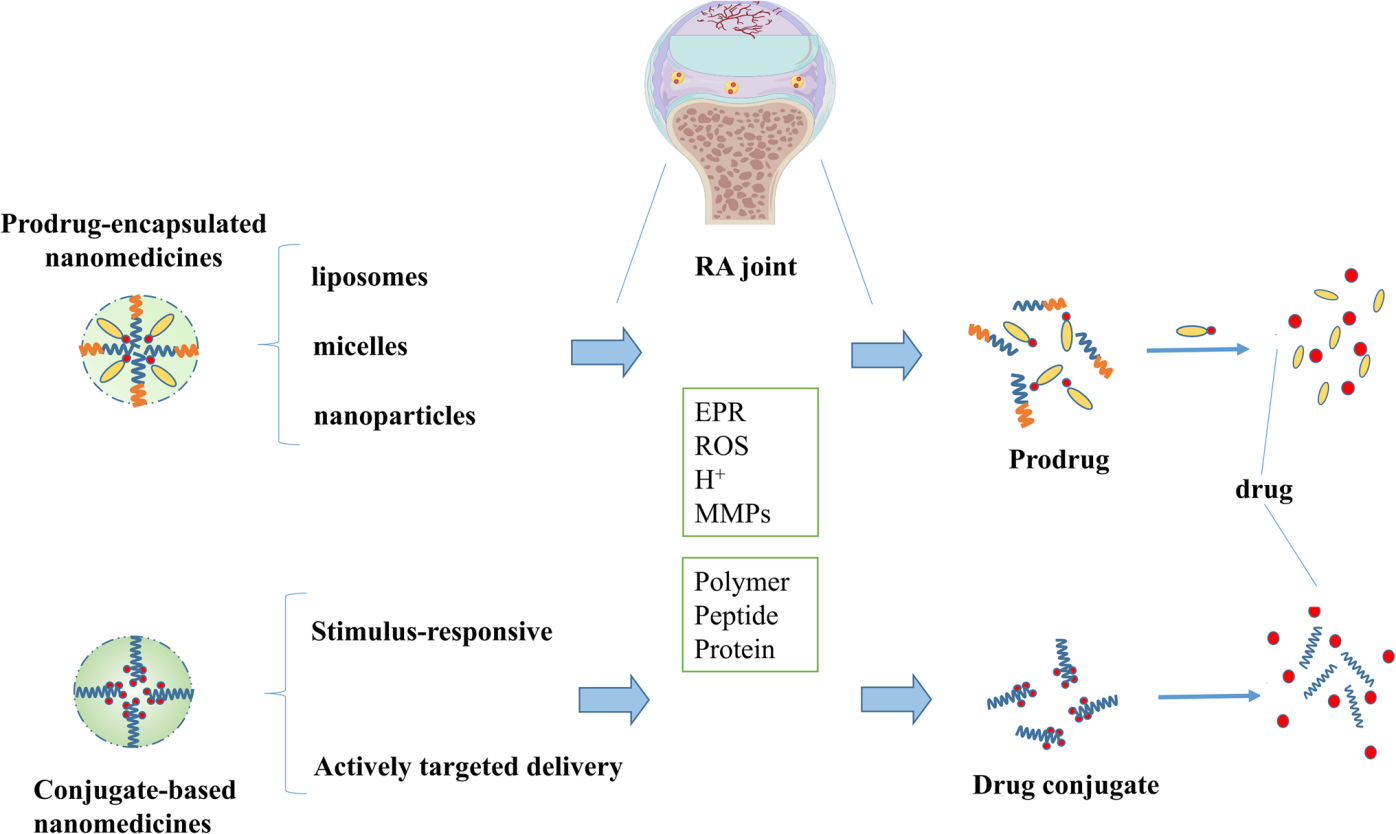
The graphical abstract of prodrug-based nanomedicines for RA

Nanocarriers attributed to the rapid development of nanotechnology, which exhibit distinct advantages in anti-tumor drug targeting delivery [30–32]. The progression of RA promotes pathological changes in RAM, resulting in changes similar to the tumor microenvironment, which provides an opportunity for nanomedicine therapy in RA. Nanomaterials are able to improve therapeutical effects and decrease adverse reactions via passive targeting (EPR) and active targeting (RAM-responsive strategies) [33, 34], making them attractive candidates for RA therapy [35, 36]. Including enhanced drug availability, facilitated accumulation of antirheumatic drugs within arthritis joints via extravasation through leaky vasculature and inflammatory cell-mediated sequestration (ELVIS) effect, precise modifications for active targeting of inflammation, and controllable drug release. However, the conventional nanoparticulate drug delivery systems (Nano-DDS) also presents a number of limitations, including low drug loading capacity, high susceptibility to crystallization during storage, serious drug leakage after administration, and toxicity due to the large amount of biological material used, the maximum achievable drug load is achieved. Prodrug-based Nano-DDS (PNDDS), which combines prodrug and nanotechnology strategy into a single system, has emerged as significant and discernible trend in recent years to conquer related limitations about conventional drug for nanomedicine therapy [37–42]. By combining these two strategies allows for better control of the chemical and biological characteristics of therapeutic drugs, preferentially target arthritic joints and illustrates the potential benefits of applying this therapeutic strategy to RA [24, 42]. For instance, nanocarriers can protect prodrugs from degradation and enable exploration of additional administration routes. Surface chemistry modifications to nanocarriers can also improve efficacy. This article presents a conclusion of prodrug-based nanocarriers for RA, containing nanosystems based on prodrug-encapsulated nanomedicines and conjugate-based nanomedicines (Table 2, Fig. 2).

**Table 2:** A summary of PNDDS used in RA treatment

Classifiction	Instance	Prodrug	Nanocarrier/conjugate	References
DMARDs	MTX	HA-MTX	HA	[43–45]
		OEGMA-MTX	OEGMA	[46]
		MTX-GDFDY	GDFDY	[47]
		MTX-HSA	HSA	[48, 49]
	Sulfapyridine	Prodrug of sulfapyridine (SP-PD)	Liposome	[50]
GCs	Methylprednisolone	Methylprednisolone hemisuccinate (NSSL-MPS)	Liposome	[51]
	Prednisolone	Prednisolone phosphate (PLP)	Liposome	[52]
	Triamcinolone	Triamcinolone acetonide21-palmitate (TAC-P)	Liposome	[53]
	Budesonide	Budesonide palmitate (BP)	Nanoparticle	[54]
	Dex	Dexamethasone palmitate (DXP)	Liposome	[55]
			Micelle	[56, 57]
			Nanoparticle	[58, 59]
		Dexamethasone phosphate (DSP)	Liposome nanoparticle	[60] [61]
		Acetone-based ketal-linked prodrug (AKP-Dex)	Nanoparticle	[62]
		HPMA-Dex	HPMA	[63–68]
		PEG-Dex	PEG	[69]
		PVP-Dex	PVP	[70]
		Anti-CD163-Dex	Anti-CD163	[71]
Biological agents	Melittin	HA-melittin	HA	[72]
NSAIDs	Diclofenac	Diclofenac ethyl ester	Micelle	[73]
Others	Artesunate	Dimeric artesunate phospholipid conjugate (Di-ART-GPC)	Liposome	[74]
		HA-TK-ART	HA	[75]
	Fumagillin	Prodrug of fumagillin (Fum-PD)	Nanoparticle	[76]
	Sinomenine	Synovial homing peptides-sinomenine	Synovial homing peptides	[77]
	ALD	TCZ-ALD	ALD antibody	[78]
	Achyranthes bidentum polysaccharide	DS-achyranthes bidentum polysaccharide	DS	[79]

## Prodrug-encapsulated nanomedicines

Vascular abnormalities and inflammatory cell infiltration are the two most notable features of RA, which are often targeted for selective drug delivery. The formation of endothelial gaps allows plasma to leak into injured areas, followed by monocyte recruitment and over activating of inflammatory factors. The constant inflammation can make blood vessels more permeable, like in solid tumors. So, the same tiny vehicles that carry anti-tumor drugs could also deliver anti-rheumatoid drugs to treat RA [80]. Passive targeting of tumors relies on the EPR effect in leaky tumor blood vessels, which allows nanoparticles to enter and remain in the tumor interstitial sites [81]. The precept is incorporated into the passive targeting nano system for RA. Inflammation induces angiogenesis at lesion site and simultaneously promotes rapid synovial sites growth through capillary-derived nutrition [82]. This process results in a wide gap of up to 700 nm between endothelial connections [83]. By means of the EPR effect, nanovehicles of suitable sizes can penetrate by the endothelial cells’ interval into synovial, where they are sequestered for sustained drug discharge. This underscores the critical role that nanovehicle size plays in passive targeting. It has been shown that the spleen is able to clear nanoparticles over 200 nm, while those less than 10 nm can pass by the filter system of kidney [80]. Additionally, macrophage is more likely to engulf larger nanocarriers compared to smaller ones [84]. In a study evaluating the treatment effect of laboratory arthritis models, nanocarriers ranging in size between 45 and 115 nm were engineered and injected [85]. The nanoparticles are 115 nm in diameter exhibited the obvious anti-inflammatory activity, indicating that appropriately sized nanocarriers enhance permeability as well as evade rapid clearance of macrophages located in the reticuloendothelial system.

As a delivery carrier for anti-rheumatic drugs, the precursor drug encapsulation nano system represents a unique nanoassembly of precursor drugs. Liposoluble prodrugs are encapsulated noncovalently in inorganic or polymeric [38]. The encapsulation of prodrugs within nano-delivery systems presents a promising approach that combines the advantages of both prodrug and nanotechnology strategy. The judicious devisal of prodrugs and the encapsulation of them into nanocarriers confers a plethora of advantages: the carrier materials and prodrugs exhibit excellent compatibility, leading to enhanced drug loading capacity, as well as decreased off-target toxicity through inflammation-specific activation of prodrugs at particular sites. Nanocarriers with passive or active targeting capabilities have been utilized for improved delivery of anti-inflammatory agents, achieving synchronous drug release via encapsulation of diverse prodrugs [62, 86, 87]. Recent developments in prodrug-encapsulated nano systems, encompassing liposomes, micelles and nanoparticles.

### Prodrug-encapsulated liposomes

Liposomes are biocompatible vesicles consisted of a phospholipid bilayer that structurally likes a cell membrane, which are widely employed as high efficiency nanocarriers for encapsulating both oleophilic and hydrophil drugs [88]. Despite methylprednisolone liposome achieved early phase success, low drug loads and premature leakage of drugs during storage are still a huge challenge. Given the above deficiency, the development of versatile lipids or chemical decoration on methylprednisolone have become a new research strategy to improve the stability and compatibility of drug encapsulation. Turjeman et al. [51] have developed PEGylated nano-liposomes that were remotely loaded with the hydosoluble amphipathic mild acid steroid prodrug methylprednisolone hemisuccinate (NSSL-MPS). Their steroidal nano-drugs with a highly efficient and stable remote loading exhibited significantly superior pharmacokinetic and biodistribution properties, as well as better tolerability and therapeutic efficacy compared to both the free pro-drugs and most drugs currently employed for treating inflammatory diseases. These findings support their further clinical development as promising therapeutic agent. Furthermore, polyethylene glycol liposomes with long-circulating characteristics were utilized by Metselaar et al. [52] to deliver prednisolone phosphate (PLP), a prodrug of prednisolone, to mice with antigen-induced arthritis (AIA). Only rats treated with PLP liposomes exhibited a remarkable recovery from weight loss. The inflammation was completely relieved within a week via single injection formula (10 mg/Kg PLP), whereas pure PLP showed only marginal efficacy. The targeting ability of PLP liposomes was demonstrated by their preferential accumulation in arthritic paws rather than healthy ones. In a separate investigation, liposomal PLP with prolonged circulation exhibited remarkable targeting efficacy towards inflamed joints [89]. Notably, treatment with liposomal PLP resulted in significant anti-inflammatory activity, leading to complete remission of paw inflammation and reduction of cartilage loss.

To mitigate the rapid drug leakage of sulfapyridine (SP) liposomes, an arthritis-responsive prodrug of SP exhibiting instability to synovial enzymes was synthesized by Kapoor et al. [50] to take advantage of the overexpression of arthritis-specific enzymes. Prodrug of sulphapyridine (SP-PD) demonstrated superior retention within liposomes in comparison to the drug alone, because combating its escape from the synovium. Biochemical estimations of various plasma and serum markers confirmed the enhanced efficacy of prodrug liposomes in mitigating inflammatory response in RA, while radiographical analysis revealed a marked reduction in soft tissue swelling following treatment with prodrug liposomes as opposed to other therapies. The utilization of bio-responsive prodrug liposomes presents a groundbreaking approach for the treatment of RA.

Ismail et al. [74] have found a new artemisinin derivative, namely a dimeric artesunate phospholipid conjugate (Di-ART-GPC), which demonstrated outstanding ability to assemble into liposomes. The Di-ART-GPC was incorporated into liposomes employing thin film dispersion-high pressure homogenization and employed as a novel therapy which is based on ART to treat anti-inflammatory for RA. Evaluations in vitro proved to have low toxicity and strong anti-inflammatory effect of the ART liposomes. Furthermore, The Di-ART-GPC liposomes showed signally higher efficacy in inhibiting the secretion of NO and pro-inflammatory cytokines by cells compared with ART alone. The in vivo evaluations demonstrated that Di-ART-GPC resulted in a reduced rate of ankle swelling and reduced inflammatory response compared to model controls and ART [90]. The results show that Di-ART-GPC liposomes have remarkable potential as a novel anti-inflammatory agent for RA.

Lopez-Garcia et al. [53] recently demonstrated the administration of liposomal triamcinolone acetonide21-palmitate (TAC-P) via intra-articular injection in a rabbit model of arthritis, and subsequently compared its efficacy to that of free TAC. Conjugating palmitate into the liposomal lipid layer significantly affected drug encapsulation. Enhanced retention in the joint cavity was observed after 8 h, while free TAC was eliminated from the joint cavity within only 1 h. Bonanomi et al. [55] have formulated liposomes loaded with dexamethasone palmitate (DXP), a prodrug of dexamethasone, ranging in size from 100 nm to 30 µm, and contrasted their efficacy to a suspension containing DXP and TAC. The anti-inflammatory effect of small-sized DXP-loaded liposomes was certified to be triple higher than that of DXP or TAC suspension, indicating their superior therapeutic potential. Furthermore, a novel and non-PEGylated liposome formulation (Micromethason) to encapsulate water-soluble dexamethasone phosphate (DSP), a prodrug of dexamethasone, was utilizedand by Pohlers et al. [60] and assessed the efficacy of liposomal DSP in rat adjuvant arthritis (AA). This novel PEG-free formulation of macrophage-targeting liposomal DSP significantly reduced the required dose and frequency for treating AA, potentially enhancing or prolonging therapeutic efficacy while also limiting side effects in RA therapy. Liposomal DSP exhibited superior therapeutic efficacy than equivalent doses of free DSP in both early and advanced stages of the disease.

### Prodrug-encapsulated micelles

Micelles are typically core–shell nanostructures composed of amphiphilic polymers, wherein the hydrophobic interactions facilitate facile encapsulation of most poorly water-soluble drugs within the hydrophobic core [91]. Moreover, it leads to an elongated circulation period and a decelerated compound release. Analogous to liposomes, the ELVIS effect plays a crucial role in drug targeting towards inflamed joints. Additionally, micelles can actively deliver drugs to specific cell types by conjugating with antibodies, sugar moieties or cell-penetrating peptides and exhibit lower complement activation than liposomes, thereby potentially reducing nanomaterial-related adverse effects [92].

Although the diclofenac loaded micelle has been ratified for rheumatism treatment, diclofenac micelles are still unstable and rapidly cleared in the blood. Due to the worst compatibilization of polymer materials with drugs, diclofenac micelles have low drug carrying capacity. Rational design of prodrugs can enhance the affinity of drug molecules to amphiphilic polymers, therefore refining the stability of colloid and drug delivery efficiency [91]. Al-Lawati et al. [73] prepared DFE-TM by co-solvent evaporation using block copolymer PEO-b-PCL, which was coupled with diclofenac prodrug ethyl diclofenac and near infrared probe cyanamide 5.5 azide. The higher fluorescence was perceived in inflamed joints of AA rats than healthy rats, indicating DFEE-TM localization in inflamed tissues because of the longer cycle characteristics of PEO following an intravenous injection. Furthermore, DFEE-TM administration for seven consecutive days significantly reduced diclofenac concentration in the hearts of AA rats compared to free diclofenac dosing. This was accompanied by a decrease in a pivotal cardiotoxicity biomarker in both heart and blood plasma. The investigation has demonstrated that the incorporation of prodrugs into polymeric micelle are supposed to effectively alleviate the toxicity of traditional treatments via modulating their biodistribution and enhancing the accumulation of pathological lesions with permeable blood vessels, such as inflamed joints [73].

**Fig. 3:**
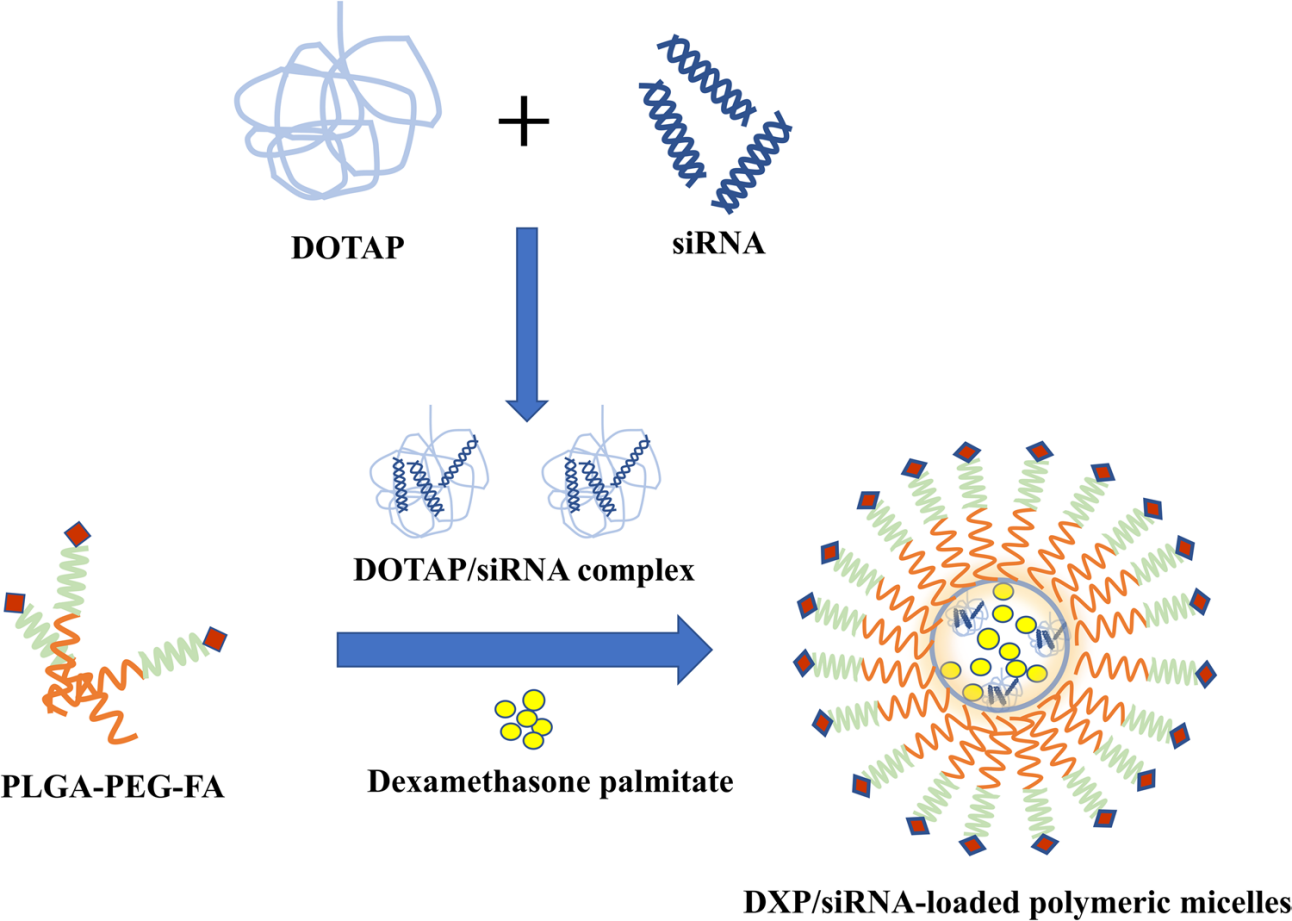
Diagram of the synthesis of DEX/siRNA-loaded polymeric micelles. Schematic illustration of siRNA/DOTAP nanocomplex followed by the encapsulation of DXP and siRNA/DOTAP nanocomplex in PLGA-PEG-based polymeric micelles. DOTAP: 1,2-Dioleoyl-3-trimethylammonium-propane

Mixed micelles probably are a superior drug delivery system owing to their EPR effect, micromolecule size, excellent in vivo stability, and facilitated preparation method compared to commercially available preparations. Egg yolk lecithin and sodium glycocholate (EYL/SGC), commonly used in micron emulsions, exhibit favorable physiological compatibility and safety profiles [93, 94]. Wang et al. [56] developed a nanopreparation utilizing mixed micelles (MMs) composed of EYL/SGC to encapsulate DXP for the treatment of RA. The mixed micelles exhibited superior bioavailability and targeting efficiency in inflamed areas, as demonstrated by pharmacokinetic analysis and in vivo fluorescence imaging. DXP-MMs intravenous injection was found to be more effective in reducing joint inflammation than the emulsion system, as confirmed by inspection of paw volume, histology and so on in an arthritis rat model induced by CFA. The Nanoscale mixed micelle consists only of a drug adjuvant, which exhibits great potential as a carrier system for lipophilic drugs in RA treatment. Activated macrophages have been found to overexpress folate receptor β (FRβ), whose binding on the nanoparticle surface can result in active targeted delivery to the tissues of RA [95]. To enhance targeting, polymeric micelles incorporating MCL-1 siRNA and DXP, which conjugated folate were successfully developed and demonstrated to be stable by Li et al. [57] (Fig. 3). The expression of MCL-1 mRNA in folate conjugated DXP/ SiRNA-supported polymer micelles (DS-FPM) was significantly lower than that of Dex/ SiRNA-supported polymer micelles (DS-FPM) or free siRNA. DS-FPM showed higher anti-inflammatory effect with the greatest decline in hind paw and lowest clinical score among all treated groups. Compared to the AIA model and free groups, DS-FPM vitally reduced the standard of TNF-α and IL-1β. The FR-targeting feature of DS-FPM demonstrates promising potential as a delivery platform for combination therapeutics (siRNA and DXP) in RA treatment.

### Prodrug-encapsulated nanoparticles

Nanoparticles possess the ability to encapsulate and deliver hydrophil drugs, lipophilic drugs, proteins and other bioactive macromolecules for targeted delivery and prolonged circulation. Xu et al. [62] synthesized eight pH-sensitive acetone-based ketal-linked prodrug nanoparticles (AKP-Dex) with varying carbon chain lengths and formulated them AKP-Dex using DSPE-mPEG200 polymer for treating RA. The compatibilization between dexamethasone and DSPE-mPEG2000 was improved by using long chain alcohols as affinity groups, and stable nanoparticles with high entrapment efficiency were formed. In a rat model of collagen-induced arthritis (CIA), nanoparticles loaded with AKP-Dex exhibited increased dexamethasone cumulation and effective release in the acid environment of inflammatory joints compared to free drugs. This article shows that pH-sensitive prodrug nanoparticles are a promising platform for treating inflammatory disorders, with enhanced efficacy and minimal systemic side effects.

Newly formed blood vessels actively participate in the inflammatory process by providing the synovial membrane of the proliferation of RA with the necessary nutrients and oxygen. Therefore, inhibition of angiogenesis can effectively interrupt the vicious cycle of inflammation and is an attractive treatment for RA [96, 97]. Aspergillin fumigin is a mycotoxin produced by Aspergillus fumigis that inhibits methionine aminopeptidase 2 (MetAP-2) thereby exerting its antiangiogenic effect. Although fumagillin was observed to be steadily integrate into the nanoparticle surfactant in vitro dissolution test, it was found to be released mainly from the lipid membrane during transport to the target in vivo [76]. Furthermore, due to its chemical instability in blood and inherent light instability during storage [98–100], fumagillin necessitates compensatory administration of higher nanoparticle doses, which collectively compromise the intended site-specific drug delivery benefits while increasing the likelihood of off-target effects. Zhou et al. [76] have developed a lipase-labile prodrug of fumagillin (Fum-PD) with Sn2 translatable moiety, which overcomes the photo-instability and enhances drug retention in circulation for improved delivery to angiogenic endothelial cells via CFDD. Targeting Sn2 fumasonicillin prodrug nanocarrier platform has been demonstrated to be efficacious in a pathological site, specifically the KRN serum-induced arthritis model. The bioactive fumagillin prodrug formulation was found to be effective, and the total dose is much lower than the previous water-soluble derivatives. The use of lipase-labile prodrugs in conjunction with lipid-based nanoparticles presents to improve the efficacy and security of fumagillin as well as other targeted nanomedicines.

To enhance dexamethasone loading and prevent its sudden release upon intravenous injection, Tsapis et al. [58] encapsulated a lipophilic prodrug of DXP within poly(DL-lactide-co-glycolide)-polyethylene glycol (PLGA-PEG) nanoparticles (NPs). DXP-loaded PLGA-PEG NPs (DXP-NPs), with a size approx. 150 nm with up to 7.5% drug load, exhibited minimal hemolytic features and cytotoxicity. DXP-NPs can suppress inflammatory cytokines in macrophages induced by LPS. After intravenous administration in mice, DXP-NPs had preferable pharmacokinetic trait than the commercial water-soluble drug DSP, with its concentration remaining elevated for up to 18 h in healthy mouse plasma. Biodistribution studies revealed that the administration of DXP-NPs resulted in lower concentrations of Dex in the liver, kidneys, and lungs compared to the soluble drug [58]. In summary, encapsulating a oleophilic prodrug of Dex into PLGA-PEG NPs presents to be a prospective strategy for improving pharmacological profiles and reducing joint inflammation in mouse models of RA. Analogously, Lorscheider et al. [59] have developed DXP-NPs stabilized by a lipid with biocompatibility and biodegradability, distearoyl-sn-glycero-3-phosphoethanolamine-N-[methoxy(poly(ethylene glycol))-2000] (DSPE-PEG2000). In a murine CIA model, these NPs demonstrated better curative effect. Passively aggregated in arthritic joints, DXP-NPs are more effective than free dexamethasone in controlling disease progression and reducing side effects [59]. Another example of Dex prodrug-based nanoparticles is that Jadhav et al. [61] prepared a Hydrophobically modified cyclodextrin-based nanoparticles loaded with dexamethasone sodium phosphate (DSP-NPs) using a double emulsion solvent evaporation method. Encapsulation of DSP resulted in a pharmacokinetic profile that showed a 2.3-fold increase in AUC and extended mean residence time, thereby enhancing the potential for nanoparticle extravasation into sites of inflammation via the EPR effect. The pharmacodynamic studies have demonstrated superior antiarthritic activity of DSP-NPs compared to marketed formulations in the AIA rat model [61]. These findings suggest that DSP-NPs hold great potential as a drug delivery system for treating RA.

Using emulsion-evaporation method, Tsapis et al. [54] designed a nano prodrug of budesonide palmitate (BP) modified by seminose. BP was synthesized through esterification, and mannosylated lipid (DSPE-PEG-Man) was obtained by reacting 1,2-distearoyl-sn-glycero-3-phosphoethanolamine (DSPE)-polyethylene glycol-amine with α-D-mannopyranosylphenyl isothiocyanate (MPITC). A lower concentration of nanoprodrug (10–10 M) was shown to be sufficient to achieve the desired anti-inflammatory effect. Overall, these findings suggest that mannosylation and budesonide palmitate accord with prodrug release and further anti-inflammatory effect of budesonide.

## Conjugate-based nanomedicines

In the case of polymer-drug conjugate-based nanomedicines, drugs are typically covalently blinded to specialized polymers through subtle chemical modifications rather than being non-covalently encapsulated within polymer nanocarriers [101, 102]. For example, an adequate dose of Dex needs to be applied to the inflamed site to exert an anti-inflammatory effect in RA. ​ However, after oral or systemic administration, Dex lacks organ-targeting capabilities, so higher doses and frequencies are required to achieve effective concentrations at the site of inflammation [103], which causes severe side effects on the hematological and alimentary systems [104]. A lot of delivery systems have been developed to ameliorate deficiencies in RA therapy, including conjugate-based nanodrugs, such as Hyaluronic acid conjugates, human serum albumin conjugates. Some of them are further modified with targeting ligands for active targeting aims.

Compared with passive targeting agents, this type of active targeting agent has a less dosage and forms higher concentrations in local tissue. In clinical application, it can reduce the occurrence of adverse reactions and improve the efficiency of drug effect. However, it is also important to note that when the drug structure changes, new adverse reactions may occur. Therefore, evaluating the safety of these prodrug-based nanomedicines is also an important factor in transitioning from basic to clinical application.

### Stimulus-responsive conjugate-based nanomedicines

Endogenous stimulation in organisms is induced by pH, enzymes, ROS etc. RA is primarily triggered by various factors, including an increase in acidic environment [105], abnormal levels of degrading enzymes [106] and the disorder of intracellular ROS [107]. So that means that this abnormal revolution can serve as specific stimulation to induce the release of therapeutic drug, which aims to manage RA in a joint purpose manner (Fig. 1).

#### pH-responsive conjugate-based nanomedicines

During RA, the lesions create a acidic environment with a pH of up to 6.0. This change in pH can be utilized as a releasing stimulation for responsive nano-DDS to regulate drug delivery [108].

According to this theory, Wang et al. [65] synthesized a novel pH-sensitive nanomedicines of Dex based on an N-(2-hydroxypropyl)methacrylamide copolymer (P-Dex). HPMA and MA-Gly-Gly-OH were copolymerized applying AIBN (2,2'-azobisisobutyronitrile) as initiator. Then the copolymerl was reacted with Boc-NHNH2 using N, N'-dicyclohexylcarbodiimide (DCC) as the coupling agent. The obtained polymer undergoes a series of reaction to obtain the hydroxypropy methacrylamide (HPMA) copolymerhydrazide conjugate. This copolymer was mixed with an excess of Dex in N, Ndimethylformamide, and one drop of acetic acid was added to catalyze the reaction, then, P-Dex was synthesized with the exemplary procedure. In rat model of arthritis, P-Dex had better and longer-acting anti-inflammatory effects than free Dex when given systemic.

To overcome the limitations of commonly used NSAIDs, GC, and DMARDs, a novel copolymer containing azides, poly (N-(2-hydroxypropyl) methylacrylamide-con-(3-azide propyl) methylacrylamide), p(HPMA-co-AzMA), has been synthesized by recently Ebbesen et al. [66]. This may provide superior polymerization properties in the field of modification flexibility and low polydispersity compared to existing RA biocoupling strategies. p(HPMA-co-AzMA) of 54 kDa display a longer circulating half-life and preferred accumulation in the inflammatory joints of mouse models of RA [66]. A HPMA–Dex conjugate with a novel structure has also been demonstrated by Liu et al. [67] and confirmed to be an successful anti-arthritis drug. This combination of well-defined polymer size and the versatility of multiple biomolecular conjugations promotes the potential application of p(HPMA-co-AzMA) in drug delivery for RA. In the similar survey, it was discovered that when the activation mechanism of P-Dex was shifted from C3 hydrazone to C21 benzoate/hydrazone linker system, drug is released more quickly, which give rise to less effective suppression of inflammation and decreased protection against joint damage [68]. Obviously, the activation mechanism of P-Dex exerts a very important effect on its pharmacodynamic characteristics. These prodrugs which are fine-tuned the activation kinetics may further promote their therapeutic efficacy and reduce the off-target side effects.

Jia et al. [63] designed and synthesized five different Dex-containing monomers with distinctively different linker chemistries with HPMA to obtain five macromolecular Dex prodrugs. Their Dex release rates were analyzed in vitro and showed extensive activation kinetics. In vivo data suggest that prodrug containing C3 hydrazone linkers is the most effective in maintaining joint structural integrity. The study also indicates that different activation mechanisms may be beneficial to identify the most effective and safest prodrugs for the treatment of RA.

Zhao et al. [64] found that aqueous solutions of polymer prodrugs were thermally responsive by adding the Dex of prodrugs to uncommonly high levels, changing from a free-flowing liquid of 4 °C to a hydrogel of 30 °C or higher. After injection of prodrug solution, hydrogel Dex was formed, which remained in the joint for more than 1 month and gradually dissolved, releasing water-soluble polymer prodrug. The prodrug is rapidly internalized by phagocytic synovial cells and processed intracellular to release free Dex, leading to sustained improvements in joint inflammation and pain in models of arthritis. Stimulus–response hydrogels have received extensive attention in a number of drug therapies [109]. Thermosensitive hydrogels are viewed to be a promising drug therapies while many polymers are temperature-responsive to phase transitions, with sol–gel transitions occurring in response to temperature changes during injection [110–112].

A HA-based pH-sensitive polymer conjugate was engineered by Shin et al. [43] for targetable drug delivery of MTX to activated macrophages with overexpressed CD44 in the inflammatory synovium. These multifunctional conjugates were produced by ester chains that lyse when exposed to the mildly acid microenvironment of the arthritic synovial membrane. HA-MTX conjugate is expected to exhibit joint tropism because of selective mutual action with overexpressed CD44 receptors on activated macrophages and synovial cells. Tamura et al. [45] has also been proved the efficacy and safety of HA-MTX conjugate. HA-MTX has a broader treatment window than oral MTX and may be a prospective agent to the therapies of RA. Wanget et al. [69] designed three kinds of PEG-Dex micelles with different Dex density. The side chain of the Dex and PEG block copolymer is connected by a derived hydrazone bond, which can be split in an acidic environment, triggering drug release. These PEG-Dex can form micelles through self-assembly and achieve targeted delivery to the site of inflammation through the ELVIS effect. By this comparative study, Wanget et al. [69] also confirmed that the optimized P-Dex had better in vivo behavior and therapeutic effect [69].

#### Enzyme-responsive conjugate-based nanomedicines

MMPs are the prototypical catabolic enzymes in arthritis. Synthesis of MMP-2 and MMP-3 is vitally risen in synovium at the periphery of hyaline cartilage in inflammatory joints [113–115]. Contrasted with other endogenic stimulation, enzyme-responsive drug delivery system offers unique advantages due to their exceptional biometric capabilities and highly efficient catalytic properties. They can also be released based on the pathological changes of disease, meaning that low disease activity will restrict unnecessary drug release and extend the treatment effect lasting-time for inflammatory arthritis [116].

A MTX-conjugated hyperbranched polymeric (HBP) nanoparticle based on oligo (ethylene glycol) methyl ether methacrylate (OEGMA) was developed by Marasini et al. [46] for examining its capability to selectively deliver MTX to inflammatory joints while saving the liver. HBPs typically have semblable chemical and structural properties to dendritic macromolecules, but are easier to synthesize and can achieve high drug loads through covalent coupling. MTX was conjugated to the hyperbranched polymer via a MMP-13 cleavable peptide linker. Expeimental results showed that in a rat model of RA, nanoparticles selectively accumulated in inflammatory joints, with a biological distribution of less than 5% in the liver after 5 days.

**Fig. 4:**
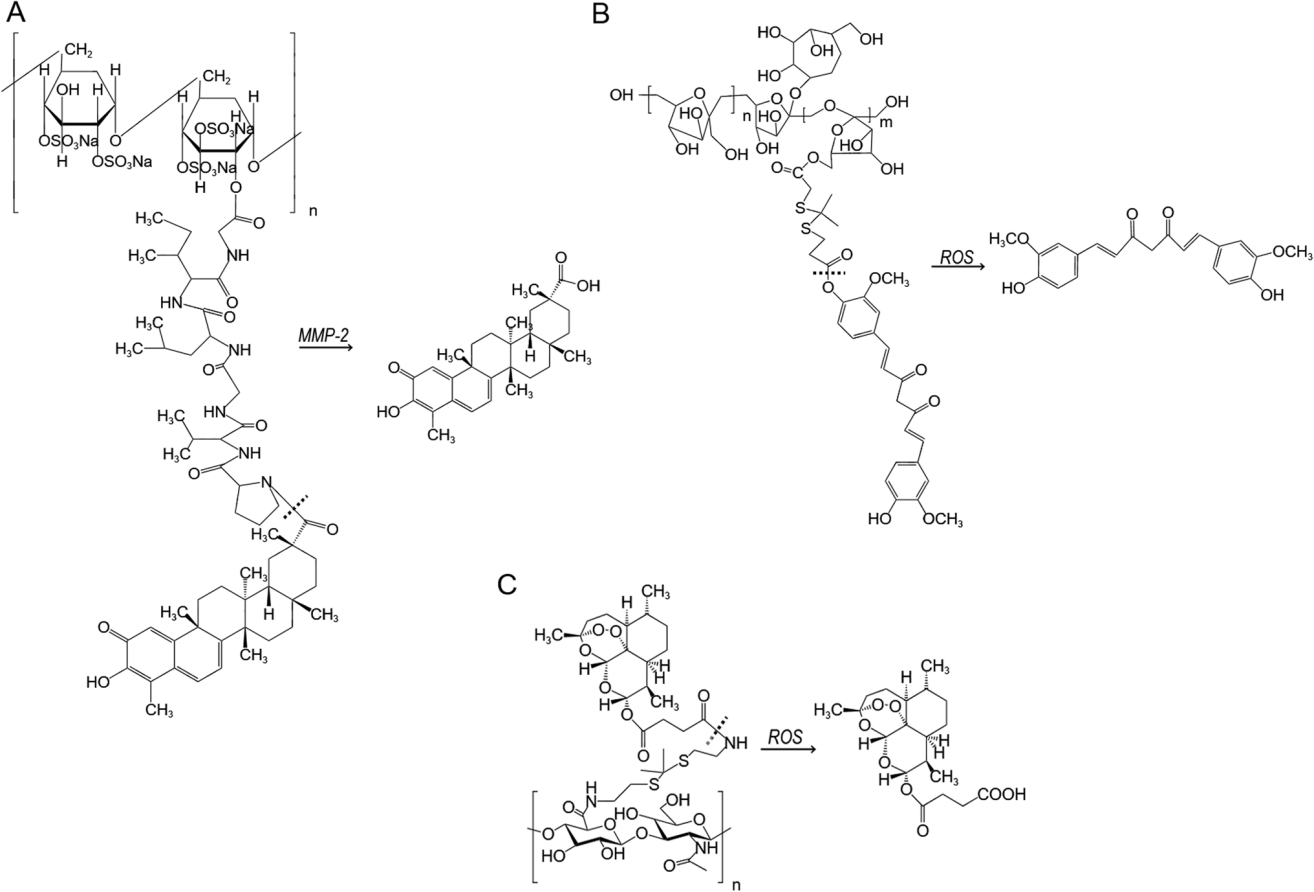
The chemical structure and activation of prodrugs (Adapted from [75, 79]). Dotted line indicates cleavage site. **A** The prodrug of DS-PVGLIG-Cel release Cel under MMP-2 response.** B** The prodrug of Abps-tk-Cur release Cur under ROS response. **C** The prodrug of HA-TK-ART release ART under ROS response

**Fig. 5:**
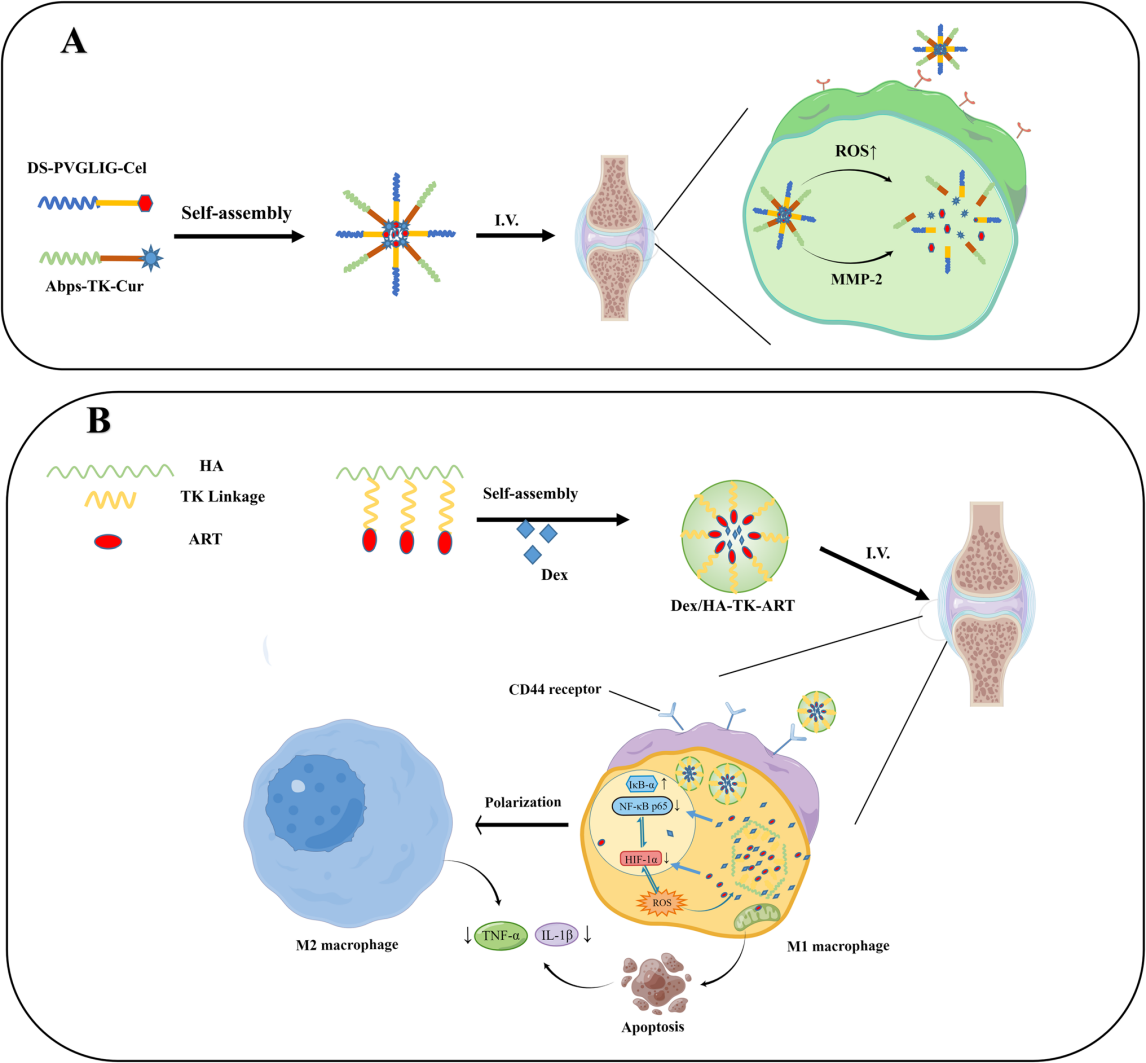
Some redox and enzyme-responsive conjugate-based nanomedicines. **A** The design with MMP-2 and ROS dual responses, and the biosynthesis schematic illustration of D&A@Cel; **B** The ROS-responsive HTA prodrug micelles for co-delivering DEX

Achyranthes bidentum polysaccharide has vital anti-inflammatory and antalgic effects, and can promote chondrocyte proliferation and suppress chondrocyte apoptosis [117]. A new self-assembled nanoparticles(DS-PVGLIG-Cel) containing Celastrol (Cel) with MMP-sensitive chemotherapy-sonodynamic therapy was engineered by Guo et al. [79] targeting macrophages at the inflammatory site of RA. Then a curcumin-prodrug nanomicelle (Abps-tk-Cur) was devised and synthesized by using Achyranthes polysaccharide as the hydrophil end and bringing into a ROS-responsive thioketal bond [79] (Fig. 4A, B). Then they mixed amphiphilic polymers DS-PVGLIG-Cel and Abps-tk-Cur prepare mixed micelles D&A@Cel. The final results showed that these bonds confer a dual response of MMP-2 and ROS nanocarriers to exert specific drug release effects within RA joints, thereby promoting the drug concentrations of triptolide and curcumin in the lesion tissue (Fig. 5A). DPC&ATC@Cel has a superior capacity to treat RA, and has an excellent safety.

#### Redox-responsive conjugate-based nanomedicines

Arthritis occurrence leads to an elevation of ROS levels not only in cells but the microenvironment. Thus, a shift of redox environments can contribute to drug release [118, 119]. The functional bonds are completely fractured under the action of redox substances, such as ROS or glutathione. These substances damage the structure of drug carrier, which is mainly composed of Boron-containing ester material, disulfide bond materials, Thioketal-containing materials and diselenide bond materials [120].

Therefore, a ROS responsive artemisinin prodrug micelle nanosystem (Dex/HA-TK-ART micelle, Dex/HTA) (Fig. 4C) was engineered by Li et al. [75] to synergically inhibit the HIF-1α/NF-κB cascade and regulate ROS clearance and macrophage repolarization in RA combination therapy. Co-released ART&Dex in response to ROS effectively performs ROS clearance and M1 to M2 macrophage repolarization by inhibiting the HIF-1α /NF-κB cascade. Intravenous injection of Dex/HTA micelles can significantly alleviate inflammatory cell infiltration and repair articular cartilage injury in AIA rat models (Fig. 5B).

### Actively targeted delivery conjugate-based nanomedicines

**Fig. 6:**
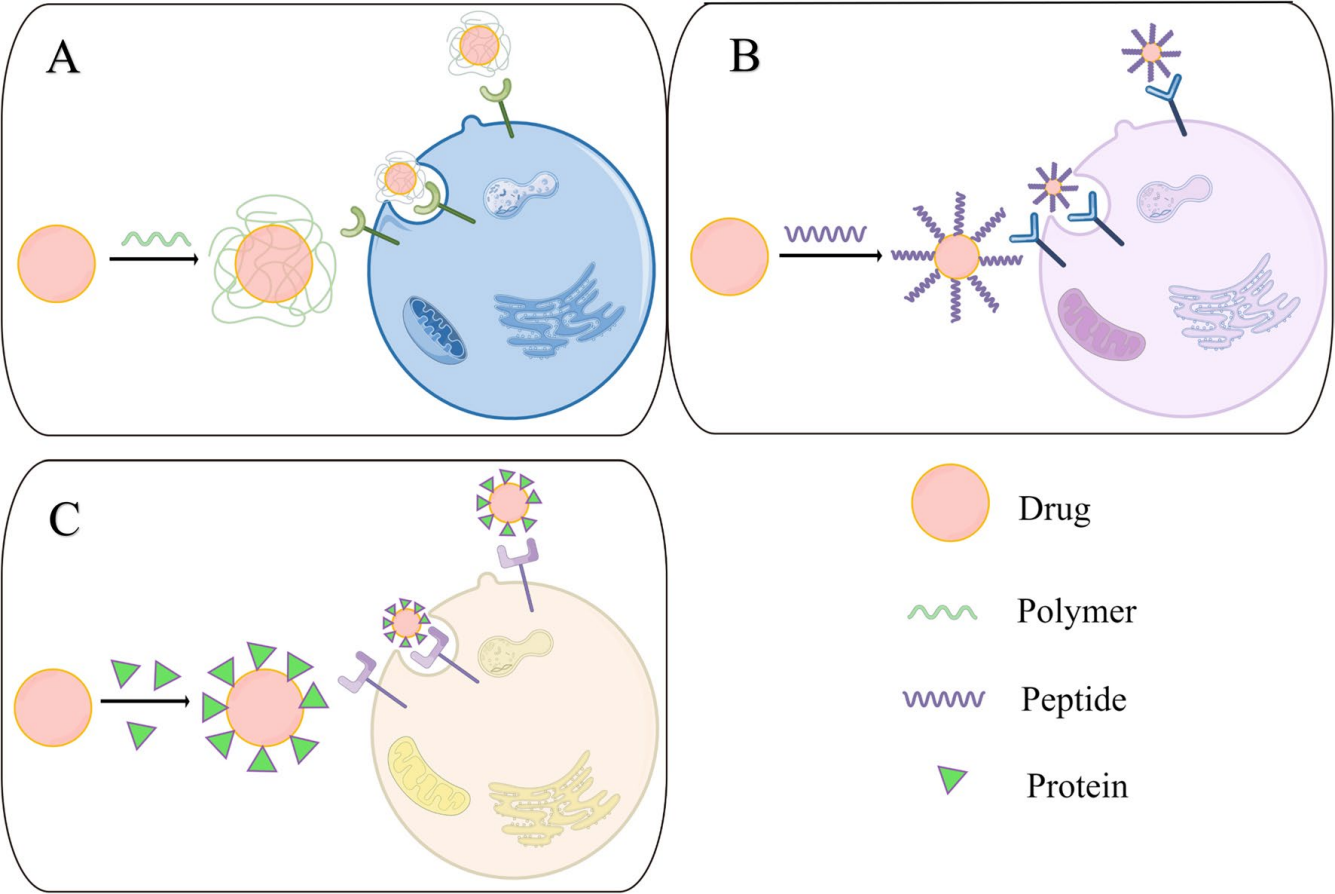
Schematic illustration of actively targeted delivery conjugate-based nanomedicines. **A** Polymer-based targeted delivery; **B** Peptides-based targeted delivery; **C** Protein-based targeted delivery

By modifying the surface of nanomaterials with appropriate polymers, peptides or ligands, it is possible to achieve both active and passive targeted drug delivery (Fig. 6).These molecules are related to disease evolution or overexpressed at sites of inflammation [121].

#### Polymer-based targeted delivery

Polymer-drug conjugates (PDCs) are supposed to be defined as the covalent connection of the pharmacologically active moieties to the polymeric chain [122]. They are commomly marked by the existence of reasonably designed covalent bonds between water-soluble polymers and bioactive molecules [123]. Attaching small molecule biologics to polymer carriers has a number of benefits, including promoted water solubility, enhanced stability, changed biological distribution and active intracellular delivery, and the potential for targeted delivery via incorporation of objective. In addition, the choice of linker chemistry has controlled the release and following activity of the drug [124] (Fig. 6A).

Hyaluronic acid (HA), the primary ingredient of the extracellular matrix (ECM), is the typical example of Natural polymers. The concentration in synovial fluid and the molecular mass of HA in patients with RA are lower than those in healthy people [125]. Thus, it was anticipated that HA may be beneficial for the management of RA. Chen et al. [126] reported that Triamcinolone (TA) with poor water solubility was conjugated with HA for intravenous injection, which increased the accumulation of definite drugs in the inflammatory arthritis of RA. The consequence of HA-TA conjugate drug showed that contrasted with osteoblast-like NIH3T3 cells treated with free TA, the apoptosis of osteoblasts was reduced, which promoted the active targeting ability of inflammatory sites and reduced the histopathological changes of arthritis.

Dextran sulfate (DS), which greatly expresses SR-A receptors on the surface of macrophages, is utilized to definitely target inflammatory tissue [127]. For instance, the prolonged administration of MTX often leads to side effects. To enhance its efficacy, Yang et al. [127] developed a DS-MTX conjugate that target activated macrophages in inflamed areas. These conjugate exhibits improved cell uptake and cumulation in arthritic joints because of the high appetency of DS for scavenger receptors overexpressed on activated macrophages.

#### Peptides-based targeted delivery

As one of the most important structural components in smart drug delivery systems (SDDs), conjugates with amino acids and peptides open new stage for the development of innovative drug delivery systems [128] (Fig. 6B).

Ma et al. [47] conjugated MTX with a supramolecular self-assembling hydrogel composed of D-amino acids with a sequence of GDFDFDY, which was 2 -naphthoacetic (Nap) acid-glycine (G) -phenylalanine (F)-phenylalanine (F)-tyrosine (Y) (termed NapGFFY). It has been found that MTX-GDFDY hydrogels exhibit a excellent behavior of drug selectivity that they increase MTX toxicity towards RA synovial cells, but decrease side effect towards normal cells. Furthermore, MTX-GDFDFDY hydrogels can not only effectively hinder the proliferation and migration of RA synovial cells, but hinder the polarization of proinflammatory M1 type macrophages to alleviate inflammation reacton. According to their findings published, MTX-GDFDFDY hydrogels successfully alleviated RA syndromes of joint redness, swelling, heat and pain compared to other hydrogels and free MTX. In addition, it significantly protects cartilage by inhibiting synovial invasion and inflammation while it does not give rise to other side effects.

Zhang et al. [77] synthesized two peptide-drug conjugates (PDCs), in which sinomenine (SIN) was connected covalently to the linear and cyclic synovial homing peptide via a 6 -aminocaproic acid linker respectively. The result indicated SIN which was conjugated cyclic form showed excellent drug effectiveness and tissue targeting and PDCs especially in its cyclic form be more effectivet for targeted drug delivery. SIN is a pure alkaloid extracted from the Chinese medicinal plant SIN, which has a wide number of biological activities, including anti-inflammatory and immunosuppressive activities [129]. For a long time, it has been clinically utilized to treat RA in China with good curative effect. SIN hydrochloride sustained-release tablets and conventional sustained release tablets are currently applied on the therapy of RA [130, 131]. Synovial homing peptides (CKSTHDRLC) have been identified to own high synovial endothelial targeting affinity.

#### Protein-based targeted delivery

Protein targeted delivery for RA therapy focuses primarily on the use of antibodies that precisely direct the drug to the cell by specifically linking to antigens on the cell [132] (Fig. 6C). Conjugations of small molecule drugs with antibodies, known as antibody drug conjugations (ADCs), are used as a new part of targeted therapeutic agents to treat cancer. ADCs not only extends the circulation time in vivo from minutes to hours, but also reduces uptake by cells through the endocytotic pathway, thereby enhancing passive delivery of drugs to diseased tissues [121].

Lee et al. [133] yet devised tocilizumab (TCZ)–alendronate (ALD) conjugate that actively targets IL-6 and blocks RA development. Within else pathogenic elements, the overexpression of IL-6 and TNF-α is the code factor that triggers the autoimmune response and inflammatory cascade in the production and development of RA [134–136]. TCZ is a antibody that specifically inhibits the function of IL-6 by linking to IL-6 receptors overexpressed in the inflammatory tissues [137, 138]. Moreover, ALD is a type of bisphosphonate, which is commonly used to prescribe osteoporosis due to its good anti-inflammatory effect [138]. Efficacy assessment of CIA mice exhibited that the TCZ-ALD conjugates had excellent efficacy on arthritis score, paw swelling, and production of proinflammatory cytokines in vivo. At the eighth week, mice fed with the TCZ-ALD conjugate never exhibited related clinical symptom with RA compared to other control group. In particular, Jonas et al. [71] developed an anti-CD163-Dex conjugate that specifically targets the glucocorticoid, Dex to the hemoglobin scavenger receptor CD163 in macrophages. The conjugate contains an average of 4Dex molecules per antibody and shows a high functional affinity for CD163. The in vivo titer of conjugated Dex was about 50 times that of nonconjugated Dex. In contrast to the strong systemic effect of nonconjugated Dex, the equivalent dose of conjugated Dex did not have this effect. Overall, this study suggests that antibody- drug conjugates are a future anti-inflammatory macrophage targeted therapy.

Patients with active RA often develop hypoproteinemia owing to high albumin consumption at the site of inflammation [139, 140]. In patients with RA, the blood-joint barrier permeability of inflammatory joints to albumin is significantly increased [141]. Albumin is a type of protein which is stable in the pH range from 4 to 9. Due to its special molecular structure, albumin has abundant functional groups and hydrophobic binding sites with high affinity, which can be used for the binding of ligands and drugs [142]. These properties and its priority uptake in inflammatory tissues, its ready availability, its non-toxicity and immunogenicity let it a significant agent in the field of drug treatment [143].

Wunder et al. [144] showed that MTX-HSA (human serum albumin) was more beneficial to MTX in restraining the generation and progress of arthritis after intra-articular administration. The results showed that albumin and MTX did not accumulate in the non- inflammatory tissues; thus, it displayed significant uptake of radio-labeled albumin, while did not exhibit significant uptake of MTX There was significant uptake of radio-labeled albumin, but not significant uptake of MTX in arthritis sites. Compared with the healthy sites, HSA of patients showed a six-fold increase in permeability of the inflamed joints. In contrast, albumin uptake in the liver and kidneys is three-fold lower than MTX. Additionally, synovial fibroblasts and activated macrophages in patients with RA absorbed fluorescently labeled albumin not only in vivo but in vitro, possibly through high expression of beta-folate receptors. With the purpose of hinder the employ of exogenous and potentially morbigenous albumin and to gain greater specific drug preparation, a MTX albumin-linking prodrug EMc-D-Alaphe-Lys-Lys (gamma-MTX) OH (EMC = 6-maleimide hexpropionic acid) has been developed by Kratz et al. [143] that rapidly binds to endogenous albumin to form a conjugate stable in human plasma. Owing to the presence of two lysine residues, it can be cleaved by hispsin B and plasminase, which are present in high levels in the synovial joints of patients with RA. In parallel, a modified form of MTX was designed by Fiehn et al. [49] to bind to endogenous albumin after intravenous administration. MTX is converted into prodrug by coupling with a polypeptide selectively bound to an endogenous albumin 34 cysteine residue. A modified form of MTX was designed to bind to endogenous albumin after intravenous administration. MTX is converted into prodrug by coupling with a polypeptide selectively bound to an endogenous albumin 34 cysteine residue.

## Conclusions and challenges

Targeted pathways are crucial for preventing chronic inflammatory diseases, reducing patient adverse reactions, and improving cure rates. Nanosystems have the potential for drug specificity and local delivery, but their biocompatibility, immunogenicity, and biodegradability are also key factors in avoiding adverse reactions such as inflammation [145, 146]. In addition, nanomaterials face a series of disadvantages during intravenous administration, such as degradation in lysosomes or other organelles. At present, most research on using nanotechnology to alleviate and treat diseases is still in the early laboratory stage. Although there are currently some liposome agents have been used in clinical practice, these drugs reduce toxicity when encapsulated by liposomes. But it is not the optimal carrier for water-soluble drugs, which also limits its use. Currently, studies have shown that modifying nanoparticles and loaded drugs can effectively enhance the active and passive targeting of drugs [147–149].

Developing nanomedicines based on prodrug design for rheumatoid arthritis is a new and very promising direction for the therapty of rheumatoid arthritis. In this article, the design principles of a series of prodrug-based nanomedicines anti-RA drugs have been summarized and discussed. RA is a chronic systemic autoimmune disease with complicated causes that can result in bone destruction, inflammation, and even disability. Despite profound progress in RA treatment, the goal of treatment is to stop or reduce inflammation, alleviate symptoms, avoid joints and organs as much as possible and restore function and overall health while minimizing long-term effects. By taking the extraordinary advantages of prodrug, containing the improvement of PK and shielding problem functional perssad, as well as the excellent drug loading capabilities, high-affinity and accurately target of nanomaterials, researchers are able to engineer optimized drug delivery systems that promote therapeutic effect, and decrease side effects of traditional drugs. The application of prodrug nanomedicines with the development of new DMARDs and biological agents DMARDs in the treatment of RA is under study, and good results have been achieved. It is believed that with the increasing clinical transformation of new preparations in the future, the treatment of RA will definitely be optimized and the progression of the disease may be inhibited.

## Data Availability

No data was used for the research described in the article.
